# Supplementary material for: Patterns of Intron Gain and Loss in Fungi
Source: PLoS Biol. 2004 Nov 30;2(12):e422. doi: 10.1371/journal.pbio.0020422 (PMC532390; doi:10.1371/journal.pbio.0020422)
Supplement: Table S1 — Also available at http://genes.mit.edu/NielsenEtAl/. (4.3 MB ZIP). [file pbio.0020422.st001.zip › NielsenEtAl/html/718.html]

AN4690.1.NCU00591.1.MG10320.1.FG08688.1


```
 CLUSTAL W (1.82) Multiple Sequence Alignments - Introns Inserted


Sequence 1: NCU00591.1	743 aa
Sequence 2: FG08688.1	714 aa
Sequence 3: MG10320.1	722 aa
Sequence 4: AN4690.1	712 aa
Alignment Length: 779 aa
Number Identitical Residues: 278 aa
Alignment Score (without introns) 14748


MG10320.1 	--MRSITKANARLP--LRHASR-------RLLSSSASASNNNNNTISQAASSLAQSITPT
NCU00591.1	--MRSIKRTSRRLPASLRAVPTSVPGLGARFLSTTAPTS-------STVTINGSTDTS--
FG08688.1 	--MRSTLRANRRLP--LKSLP--------RFLSTTAPSS-------ASTPSVASSSPTPK
AN4690.1  	MPLSSLLRTSARLG-----------------QTVVARRS-------RRTASTVTSNSSSN
          	 .: *  ::. **                   :  *  *         ..   : . :..

MG10320.1 	TTPITSLLIANRGEIAL~RIHRTATRLGIPTTTVFTDVDRHSQHAAASPSSIALGSDPRA
NCU00591.1	VTPISSVLIANRGEIAL~RVARTAASMGIRTTTLYTDIDAASQHAKCTPYALSLG-APSA
FG08688.1 	QTPISSVLIANRGEIAI~RINRTAERLGIRATTVYTDIDAGSWHASSGFQSLGLG-PANA
AN4690.1  	FRALDSILIANRGEIAL2RVGRTAAQHGIRVTTLYTDPDSQAQHALSTPYAFNLG-SVSA
          	  .: *:*********: *: ***   ** .**::** *  : ** .   :: **    *

MG10320.1 	YLDGDRISRLAADKLGPGVALHPGYGF~LSENAAFARLCADRGVNFVGPPASAMADMGDK
NCU00591.1	YLDGPRIVELAKQHG--IQALHPGYGF~LSENPAFAKACEEAGIVFVGPPAKAMTDMGDK
FG08688.1 	YLDGEKIISLAKQNG--IQALHPGYGF~LSENSKFAERCEEEGIVFVGPPATAMADMGHK
AN4690.1  	YLDGDRIIEIAKAQG--CQGIHPGYGF0LSENSEFARKCTEAGLVFIGPPWKAIEDMGDK
          	**** :*  :*  :     .:****** ****. **. * : *: *:*** .*: ***.*

MG10320.1 	A~RSKHIMSAAGVPCVPGYHGEEQSAAFLAEKAAEIEYPVLLKSVKGGGWKGD-------
NCU00591.1	A~RSKEIMNASGVPCVPGYHGSEQSVEQLKAKAADIKYPVLLKSVKGGGGKGMRIVRNAD
FG08688.1 	A~RSKEIMTAANVPCVPGYHGADQGEQELLEHAKKITFPVLLKSVRGGGGKGMRIVLTEE
AN4690.1  	S2QSKHIMTAAGVPCVPGYHGENQDPNFLEAEADKIKYPVLIKAIKGGGGKGMRIARSKE
          	: :**.**.*:.********* :*.   *  .* .* :***:*:::*** **     . .

MG10320.1 	-------------------------------AHRRVGGR0VFADRHGGCVALGERDCSIQ
NCU00591.1	EFEAQLKSARAEARASFGEGGEVMLVEKYIVRPRHVEVQ~IFADRHGNCVALGERDCSVQ
FG08688.1 	EFLTQLRSARAEAKASFGEGGEVMLVEKYIIRPRHVEVQ~VFADKWGNTVALGERDCSIQ
AN4690.1  	EFQAQLQSAKSEAMNSFG--DDHVLVEKYITTPRHIEVQ~VFADKHGNCVALGERDCSIQ
          	.  :.  :: :.:  : .  ..    ..     *::  : :***: *. *********:*

MG10320.1 	RRHQKVIEESPAPGLSEELRQDLWAKARTAALAVGYVGAGTVEFILDRDTDRFYFMEMNT
NCU00591.1	RRHQKILEESPAPLLDDVTRKDLWDKARKAALAVDYVGAGTVEFILDKDTNEFYFMEMNT
FG08688.1 	RRHQKILEESPAPDLDLATRHDLWDKARKAASAVGYVGAGTVEFILDKDTNKFYFMEMNT
AN4690.1  	RRHQKILEESPAPHLPDATRKDIWAKARSAALAVRYEGAGTVEFIFDNDTGEFFFMEMNT
          	*****::****** *    *:*:* ***.** ** * ********:*.**..*:******

MG10320.1 	RLQVEHPVSEMVTGTDLVEWQLRVAAGERLPMTQAEIEQKIAERGWAIEARIYAENPEKG
NCU00591.1	RLQVEHPVSEMVTGTDLVEWQFRVAAGEKLPLTQGEIEARILERGAAIEARIYAENPEKG
FG08688.1 	RLQVEHPVTEMVTGLDLVEWQFRVAAGEKLPLSQEEVETQMNERGAAIEARIYAENPEKG
AN4690.1  	RLQVEHPVTEMVTGQDLVHWQLKVAEGAELPLTQEEVEANIATHGHAIEARIYAENPAQG
          	********:***** ***.**::** * .**::* *:* .:  :* *********** :*

MG10320.1 	FMPDSGKLVHLVTPKTDTEVHRPEDAAVRIDAGFVEGDTVSQAYDGMIAKLIVRGADRET
NCU00591.1	FFPDSGTLV--HLSKP------QTNEDVRIDAGFVEGDTVSEAYDGMIAKLIVRGKDRET
FG08688.1 	FIPDSGKLVRAYLPAE------LQNEDVRLDWGFRSGNTISEAYDGMIAKLIVRGDTRER
AN4690.1  	FIPDSGALLHVRTPAT--------TEDVRIDAGFVQGDEVSAHYDPMIAKLIVRGADRQE
          	*:**** *:    .             **:* ** .*: :*  ** *********  *: 

MG10320.1 	AIKRMELALREYEVVGLSTNIEFLKRLCRSPAFIQGDLDTGYIDRWREELFQPRRVEDEV
NCU00591.1	AIRKLELALREYEVVGLSTNIEFLKRLCNSQAFIEGDVETGFIDKWKTELFEAKPTSNDV
FG08688.1 	AIAKMESVLRSYEIVGVATNIEFLKRLCETDAFVEGDVETGFIDKWREQLFKPRPIKNEV
AN4690.1  	AIRKLAAALEEYEVAGPATNIEFLKSVCKSADFISGKVETGYIEKHHDELFAQSPIEPEV
          	** ::  .*..**:.* :******* :*.:  *:.*.::**:*:: : :**     . :*

MG10320.1 	FVQAALGLLAPVKQTVQQMGLGAHGETLGFGEAAASTGERRFAFKVTDGYAEQAAEGKGE
NCU00591.1	FAQAALGLLG----TQTQNVPAAHGQALGFGEATT-QNSRKFAFRVQDDASTEEGK----
FG08688.1 	VAQAALGMVN----SELQNS-GPHGLTLGFGEANS-IGERKLSFKIQDGYSQEEGE----
AN4690.1  	LAQVALACLHDDARLAAQKATNFQGSAVGFGPGFQ---QHHMTFTNSASANNEAFD----
          	..*.**. :        *     :* ::*** .     .::::*    .   :  .    

MG10320.1 	DVVEVTLTQRGNNLYNARVARRGEQTPIEFDNVTSEP-----TESSDLKTKVKSFFPLSR
NCU00591.1	-IVEVEVAQLNNKLFTVSVRKEGDDKPQVFENIVSKSTSTTAPTSSAKKTKLTTYFPLAR
FG08688.1 	-VVEASVTQVAHNLYNVSISRKGNETPQVFTNIACK------PEPEGEVMKLESYFPFER
AN4690.1  	----VKVQQTGENVFNVKIG------EHTFEQVTSHP--------NADFRIITSFFPHTR
          	    . : *  .:::.. :          * ::....        .     : ::**  *

MG10320.1 	VESTVVRDPINSD-KITVFQLGTKTDLSLVQPGWFDKALGLKEAAASVVAPMPCRIMRVE
NCU00591.1	VDTTLVQDPADNE-KLTVFQLGHKTQLRLVSPVWFDKALGLKEVAASVVAPMPCKILRNE
FG08688.1 	IQSSVVPQHTDNDTKVTVFQHGVKTDLVLLPPKWYEKALGLKESSASVAAPMPCKILKNE
AN4690.1  	LDTTVIRD---GD-SIVVFQRGRQYRLTTPRAKWMEKALGMKDVTNSVLAPMPCKVLRVE
          	:::::: :   .: .:.*** * :  *    . * :****:*: : ** *****:::: *

MG10320.1 	VAEGDSVEKGTPLVV2IESMKMETVIRSPQAGVVKKLAHKEG~DICKAGTVLVLFEEDAA
NCU00591.1	VEEGQPVEKGAPLVV2IESMKMETVIRSPQSGVIKKLAHKEG0DICKAGTVLVTFEETEE
FG08688.1 	VEEGQTVQKGAPLVV2KT--------------------NRLL0DICKAGTVLVMFEEAEG
AN4690.1  	VQAGDTVEKDQPLVV~IESMKMETVIRSPQRGKIAKVVHQKG0DQCKSGTPLVEFAGEDE
          	*  *:.*:*. ****   : . .:   :.. .   .  ::   * **:** ** *     

MG10320.1 	EQS--
NCU00591.1	SA---
FG08688.1 	KDGDA
AN4690.1  	EASK-
          	. ..
```
